# Post-traumatic stress disorder and social determinants of health among individuals with cancer: association with healthcare utilization and outcomes in the All of Us Research Program

**DOI:** 10.1007/s11764-026-01993-8

**Published:** 2026-03-03

**Authors:** Sebastian O. Ekenze, Odysseas P. Chatzipanagiotou, Azza Sarfraz, Qaidar Alizai, Andrea Baldo, Timothy M. Pawlik

**Affiliations:** Department of Surgery, The Ohio State University Wexner Medical Center and James Comprehensive Cancer Center, Columbus, OH USA

**Keywords:** Posttraumatic stress disorder (PTSD), Cancer survivorship, Social determinants of health (SDoH), Healthcare utilization, Quality of life

## Abstract

**Background:**

Individual effect of posttraumatic stress disorder (PTSD) and social determinants of health (SDoH) on healthcare utilization and well-being among cancer patients remains unclear. We sought to assess independent and combined effects of these factors on healthcare utilization and self-reported well-being.

**Methods:**

All of Us Research Program was used to identify participants with cancer and/or PTSD, as well as individuals with neither. SDoH burden, healthcare utilization, and well-being were assessed relative to PTSD on multivariable logistic regression adjusting for sociodemographic factors.

**Results:**

Among 16,261 participants, 11.6% (*n* = 1895) had cancer, 1.4% (*n* = 228) had cancer and PTSD, and 20.1% (*n *= 3281) had PTSD alone. PTSD was associated with higher odds of emergency room visits (aOR 4.16, 95% CI 3.80–4.55), poor mental (aOR 3.77, 95% CI 3.43–4.14), and emotional health (aOR 3.76, 95% CI 3.41–4.16) (all *p* < 0.001). High SDoH burden independently predicted increased inpatient and emergency visits (aOR 1.24, 95% CI 1.13–1.36), as well as greater risk of poor quality of life (aOR 3.71, 95% CI 3.20–4.32) (all *p* < 0.001). Cancer patients with PTSD and high SDoH burden had substantially greater odds of poor mental health (aOR 10.92, 95% CI 9.33–12.77) and poor emotional health (aOR 12.20, 95% CI 10.33–14.41) (all *p* < 0.001).

**Conclusion:**

Cancer, PTSD, and high SDoH burden were independently associated with greater healthcare use and worse well-being with combined exposures amplifying risk among cancer survivors.

**Implications for cancer survivors:**

Survivors with PTSD and high SDoH burden are especially vulnerable. Integrating trauma-informed care and SDoH screening strengthens survivorship equity and outcome.

**Supplementary Information:**

The online version contains supplementary material available at https://doi.org/10.1007/s11764-026-01993-8.

## Introduction

Cancer remains an important global public health challenge with substantial physical, psychological, and socioeconomic burdens on patients and healthcare systems [1–3]. Beyond the direct impact of the disease and its treatment, a cancer diagnosis can act as a traumatic event triggering psychological distress that, for some patients, meets criteria for posttraumatic stress disorder (PTSD) [3–5]. PTSD in cancer patients manifests through intrusive recollections, avoidance, negative alterations in mood and cognition, as well as hyperarousal, and may persist long after treatment completion [4, 6]. It is estimated that 4–5% of cancer survivors experience PTSD, with even higher rates of subthreshold symptoms [6, 7]. Subthreshold PTSD symptoms, though not meeting full diagnostic criteria, may progress to full PTSD among cancer survivors [8, 9]. PTSD and subthreshold PTSD among cancer patients have been reported to cause significant distress and impair function, as well as possibly increase healthcare use among cancer survivors [8, 9].

Despite its clinical significance, PTSD in cancer populations often goes undetected or untreated [10]. In the USA, only about one-half of individuals with PTSD access care, and even fewer receive treatment from a mental health professional [11, 12]. Reported barriers include high out-of-pocket costs, limited availability of specialized providers, and stigma surrounding mental health care [11, 12]. Overall, the societal cost of PTSD among the civilian population has been estimated at $189.5 billion annually, encompassing direct medical expenditures, lost productivity, unemployment, and other indirect costs [12]. For cancer patients, these costs may be compounded by intensive and prolonged treatment needs, frequent follow-up care, and surveillance for recurrence [13].

Social determinants of health (SDoH)—defined by the U.S. Department of Health and Human Services as “conditions in the environments in which people are born, live, learn, work, play, worship, and age” that influence health and quality-of-life outcomes—are increasingly recognized as critical drivers of cancer outcomes [14–16]. Adverse SDoH such as food and housing insecurity, unemployment, lack of transportation, healthcare access barriers, and discrimination can delay diagnosis, limit treatment options, and worsen survival and survivorship outcomes [15–18]. Importantly, these factors can operate independently of disease biology, exerting effects that rival or exceed the direct impact of cancer [19, 20].

The relationship between PTSD and SDoH is complex and bidirectional. Persistent SDoH stressors can exacerbate PTSD symptoms, while PTSD may impair socioeconomic functioning, increasing social vulnerabilities and restrict access to resources [20–22]. Recent analyses using the National Institutes of Health All of Us Research Program demonstrated that adverse SDoH account for a substantial proportion of healthcare utilization and decrements in self-rated well-being associated with PTSD in civilians, often exceeding the contribution of PTSD itself [22]. These relationships have not been examined in cancer populations, among whom treatment intensity and prolonged survivorship stress may amplify the combined effects of cancer, PTSD, and social determinants of health on healthcare utilization and patient-reported well-being. Prior studies in general or veteran populations have demonstrated that domains such as employment status, affordability of care, discrimination, and social support are particularly influential in shaping health outcomes [23, 24]. To date, no study has quantified these effects specifically among cancer patients with and without PTSD.

We conceptualized cancer, posttraumatic stress disorder (PTSD), and social determinants of health (SDoH) as interrelated factors associated with healthcare utilization and patient-reported outcomes. A cancer diagnosis and its treatment experience may constitute a traumatic stressor, with PTSD manifesting through intrusive recollections, avoidance, negative alterations in mood and cognition, and hyperarousal that may persist long after treatment completion. PTSD may be further associated with functional impairment and interact with adverse SDoH, while SDoH can influence access to care, healthcare engagement, and quality of life and other patient-reported outcomes. Together, these interrelated factors are hypothesized to be associated with increased healthcare utilization and poorer self-reported outcomes (Supplementary Fig. 1).

Therefore, the objective of the current study was to evaluate the independent and combined effects of a cancer diagnosis, PTSD, and social determinants of health (SDoH) relative to healthcare resource utilization (HCRU) and patient-reported outcomes in a national cancer cohort. We hypothesized that adverse SDoH would substantially—and in many cases independent of PTSD—drive greater healthcare use and worse well-being among cancer survivors. Understanding these independent relationships may be critical to design equity-focused interventions that address both the clinical and social needs of cancer patients, ultimately improving outcomes and reducing disparities in oncology care.

## Materials and methods

### Data source, study population, and cohort selection

Data from the All of Us database were used to obtain a comprehensive view of an individual’s overall health and well-being by longitudinally integrating electronic health records (EHR), genetic information, lifestyle behaviors, environmental exposures, and a broad range of social determinants of health [25, 26]. As of August 2025, the All of Us database had over 600,000 participants contributing survey, EHR, biometric, genomic, and wearable data. Responses were collected from 582,671 participants, 259,189 of which were on SDoH; the EHR indicated a diagnosis of PTSD in 21,409 individuals and a documented history of primary malignant tumor of the breast, lung, colon, rectum, prostate, liver, biliary tract, pancreas, urinary bladder, kidney, thyroid, or uterus in 24,610 patients. These cancers were chosen to maximize clinical relevance and provide sufficient consistency and power [27] to evaluate the effect of PTSD, cancer, and SDoH on healthcare utilization and self-reported well-being. The program’s recruitment approach and underlying scientific goals have been previously reported [28].

**Fig. 1:**
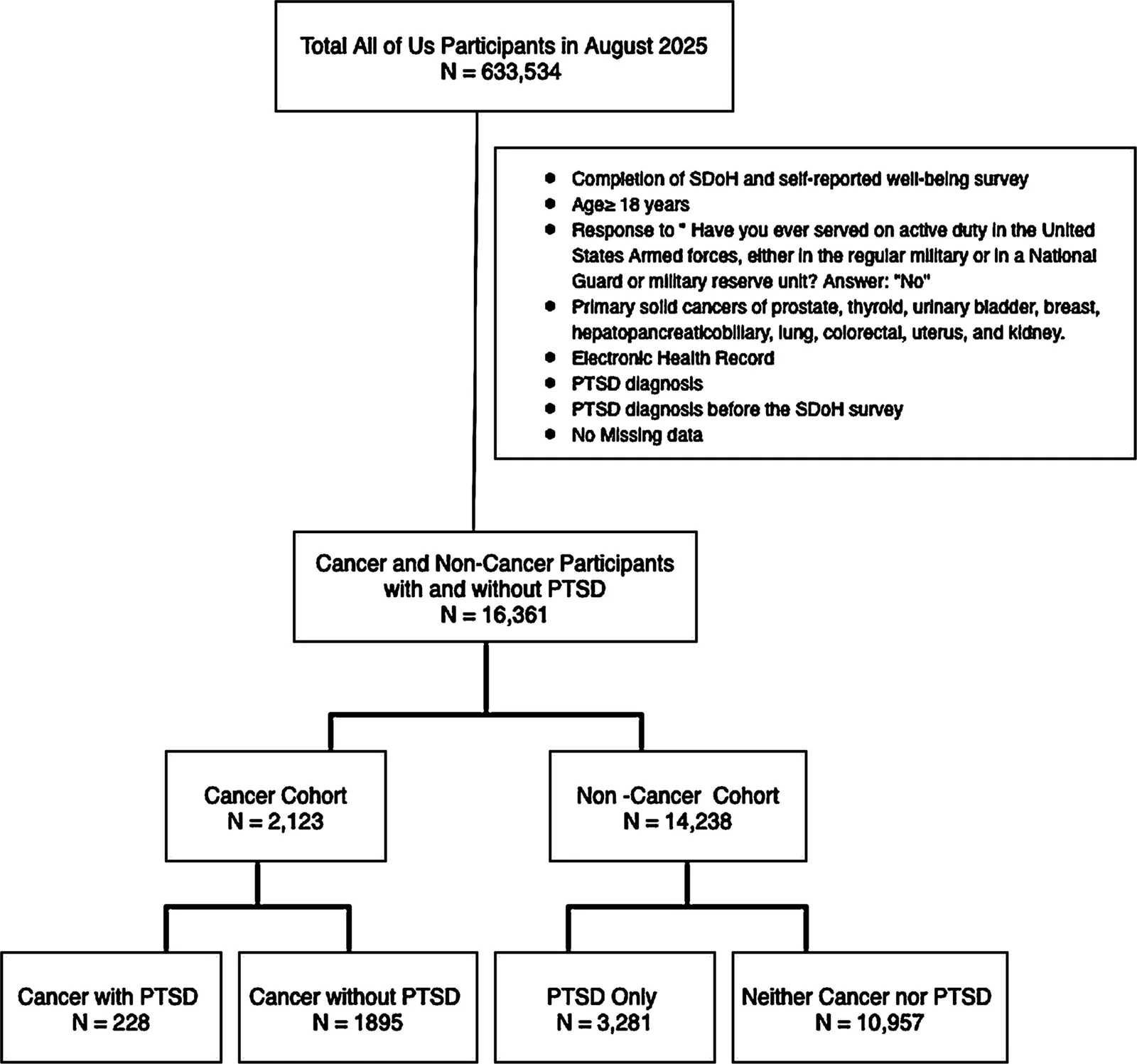
Selection process leading to the final study populations. Bullet points represent study inclusion criteria

From the All of Us Research Program, adults (≥ 18 years) who completed the SDoH and self-reported well-being survey, had available EHR data, had PTSD diagnosis before survey completion, were non-military, and without missing data were included in the analytic cohort. Participants were excluded if younger than 18 years, had multiple primary tumors, or were missing data on SDoH and self-reported well-being survey or had diagnosis of PTSD after the date of the survey. Participants were classified into four mutually exclusive cohorts based on cancer and PTSD status: (1) cancer plus PTSD, (2) PTSD alone, (3) cancer alone, and (4) neither cancer nor PTSD (control). Each cohort was separately exported to the All of Us R virtual environment to create four parallel datasets that differed only by cohort membership; these cohorts were combined into a single analytic dataset for all analyses. Participants provided informed consent, with NIH IRB approval obtained, and all authorized users followed Common Rule principles outlined in the Belmont Report [28]. Figure 1 illustrates the selection process leading to the final study population.

### Main exposures

Cancer and PTSD status were ascertained from electronic health records (EHR) using standardized diagnostic codes. Participants were categorized into four mutually exclusive groups: (1) cancer with PTSD, (2) cancer without PTSD, (3) no cancer with PTSD, and (4) neither cancer nor PTSD. Social determinants of health (SDoH) were measured using survey responses to questions covering economic stability, education access and quality, healthcare access and quality, neighborhood and built environment, and social and community context (Supplementary Table S1), as defined by the Healthy People 2030 framework [14]. Survey responses were re-coded on a numerical scale from 1 to *n* (number of response options), with 1 indicating the most unfavorable health determinant. Item scores were aggregated into standardized domain scores across the five SDoH domains. Each domain score was dichotomized at the sample median with values below the median classified as high burden (1) and individuals at or above the median as low burden (0). An overall SDoH score was computed by combining the five domain scores and similarly dichotomized at the median into high SDoH burden (1) and low burden (0) [26].

### Outcomes

Study outcomes were grouped into healthcare utilization and self-reported well-being.

Healthcare utilization outcomes included outpatient visits, emergency room visits, inpatient visits, ambulatory visits, and other visit types (home, telehealth, laboratory, case management, and pharmacy). Each was coded as binary (1 = present, 0 = absent) based on their utilization in 2022 and 2023. Self-reported well-being outcomes included survey responses to general health, quality of life, physical health, mental health, social satisfaction, fatigue, and emotional problems (Supplementary Table 2). General health, quality of life, physical health, mental health, and social satisfaction were assessed on a 5-point scale (excellent, very good, good, fair, poor) and recoded into binary variables: poor (fair/poor = 1) versus good (excellent/very good/good = 0). Fatigue was rated as none, mild, moderate, severe, or very severe and recoded as poor (moderate/severe/very severe = 1) versus good (none/mild = 0). Emotional problems were rated as never, rarely, sometimes, often, or always and recoded as poor (sometimes/often/always = 1) versus good (never/rarely = 0) [29].

### Covariates

Sociodemographic variables included age, sex assigned at birth, race/ethnicity, marital status, education, employment status, income, and health insurance coverage. Age was calculated based on participant birthdates and the date of overall health and social determinants of health survey completion. Race/ethnicity was categorized as Non-Hispanic White, Non-Hispanic Black, or Other (including Hispanic, Asian, American Indian, and Alaska Native) to ensure that each category had ≥ 20 participants. Similarly, marital status was classified as married/living with partner, never married/widowed, or divorced/separated; and educational attainment was categorized as less than college, some college, or college graduate or higher. Employment status was classified as employed/self-employed, or unemployed. Health insurance coverage was categorized into insured or uninsured [28].

### Statistical analysis

Demographic characteristics and SDoH burden were summarized by cohort groups (cancer with PTSD, cancer only, PTSD only, and control group) using descriptive statistics. Continuous variables were summarized as means with standard deviations (SD), and categorical variables as frequencies with percentages. Group differences were evaluated using ANOVA for continuous variables and chi-squared or Fisher’s exact tests for categorical variables, as appropriate. Separate binary indicators for PTSD (yes/no) and cancer (yes/no) were created from the merged dataset and, in conjunction with the SDoH burden (yes/no), were used to determine the associations of PTSD, cancer, and SDoH with healthcare utilization and self-reported well-being outcomes. These were initially examined using univariable logistic regression with PTSD (no) and cancer (no), as well as SDoH low burden as references. Based on outcome distributions across exposure groups (Supplementary Table 3), outpatient visits were excluded due to insufficient variation (99.9% had ≥ 1 visit; *n* = 21 had none) and ambulatory visits due to very low counts in some groups. For the current study, acute healthcare utilization encompassed emergency department visits and inpatient admissions, representing unscheduled or high-intensity care; however, each outcome was analyzed separately to reflect differences in clinical context and utilization patterns [22]. A *p*-value < 0.05 was considered statistically significant. To estimate the independent effects of PTSD, cancer, and SDoH on the outcomes, multivariable logistic regression models adjusting for age, sex at birth, race/ethnicity, education, employment status, income category, health insurance coverage, and marital status were used. To assess interaction and joint effects of PTSD, cancer, and SDoH, each binary outcome was modeled using logistic regression incorporating main effects for PTSD and cancer, their interaction (PTSD × cancer), SDoH burden, and the covariates. To estimate joint effects, multivariable logistic regression models were fitted that included main effects for each exposure, any interaction term, and covariates. The general model was specified as:

$$\mathrm{Logit}\{P(Y=1)\}=\beta0+\beta1\mathrm X1+\beta2\mathrm X2+\beta3(\mathrm X1\times\mathrm X2)+\sum k\;\gamma\mathrm{kZk}$$

where *Y* denotes the binary outcome, X1 and X2 represent binary exposure indicators (e.g., PTSD [yes/no] and cancer [yes/no]), X1 × X2 is their interaction term, and Zk denotes covariates. The adjusted odds ratio (aOR) for the joint exposure (e.g., PTSD = 1 and cancer = 1) relative to the reference group (PTSD = 0 and cancer = 0) was calculated by exponentiating the linear predictor corresponding to both exposures being present:

$$\mathrm{aORPTSD} + \mathrm{Cancer}=\mathrm{exp}\,(\beta 1 + \beta 2 + \beta 3)$$

Similarly, joint effects for cancer and high-burden SDoH were calculated as:

$$\mathrm{aORCancer} + \mathrm{SDoH}=\mathrm{exp}\,(\beta \mathrm{Cancer} + \beta \mathrm{SDoH}+ \beta \mathrm{Cancer} \times \mathrm{SDoH})$$

And joint effects for PTSD and high-burden SDoH were calculated as:

$$\mathrm{aORPTSD} + \mathrm{SDoH}=\mathrm{exp}(\beta\, \mathrm{PTSD} + \beta \mathrm{SDoH} + \beta \mathrm{PTSD} \times \mathrm{SDoH})$$

Interaction *p*-values were used to test departure from multiplicativity on the odds ratio scale, consistent with established methods [30]. Multivariable logistic regression was also used to estimate the independent association of each SDoH domain with the study outcomes, adjusting for all other SDoH domains and demographic covariates. Adjusted odds ratios (aORs) with 95% confidence intervals (CIs) were reported for all parameters. All tests were two-sided, and analyses were conducted within the secure All of Us Researcher Workbench using R version 4.3 (R Foundation for Statistical Computing).

## Results

### Baseline cohort characteristics

Among the 16,361 All of Us participants included in the analytic cohort, 2123 (13.0%) individuals had a primary solid cancer involving the prostate (*n* = 1501, 70.7%), thyroid (*n* = 125, 5.9%), urinary bladder (*n* = 91, 4.3%), breast (*n* = 88, 4.1%), hepatopancreaticobiliary (*n* = 81, 3.8%), lung (*n* = 80, 3.8%), colorectal (*n* = 76, 3.6%), uterus (*n* = 43, 2.0%), or kidney (*n* = 38, 1.8%). Overall, 228 individuals had cancer and PTSD (prostate cancer (*n* = 156, 68.4%), thyroid cancer (*n* = 17, 7.5%), hepatopancreaticobiliary cancer (*n* = 11, 4.8%), lung cancer (*n* = 10, 4.4%), breast cancer (*n* = 9, 3.9%), uterine cancer (*n* = 9, 3.9%), colorectal cancer (*n* = 7, 3.1%), bladder cancer (*n* = 5, 2.2%), and kidney cancer (*n* = 4, 1.8%)). There were 3281 individuals with PTSD only. Mean age of participants was 62.4 years (SD = 14.5); the majority was male (*n* = 13,067, 79.9%) and identified as non-Hispanic White (*n* = 12,601, 77.0%). More than one-half of individuals had a college degree or higher (*n* = 8955, 54.7%); nearly all participants were insured (*n* = 15,717, 96.1%), and one-third reported annual household income > $100,000 (*n* = 5446, 33.3%). Most individuals were married or living with a partner (*n* = 10,741, 65.7%). Roughly one-half of the cohort had a high SDoH burden (*n* = 7762, 47.4%) (Table 1).

**Table 1:** Baseline socio-demographic characteristics of study participants by cohort groups

Socio-demographic characteristics	Overall (*n* = 16,361)	No cancer no PTSD (*n* = 10,957)	PTSD only (*n* = 3281)	Cancer only (*n* = 1895)	Cancer and PTSD (*n* = 228)	*p*-value
Age (mean, SD)	62.38 (14.49)	62.92(14.30)	54.36(13.78)	72.31(8.74)	69.53 (8.36)	< 0.001
*Sex at birth*, *n* (%)						< 0.001
Female	3294 (20.1)	2273 (20.7)	820 (25.0)	172 (9.1)	29 (12.7)	
Male	13,067 (79.9)	8684 (79.3)	2461 (75.0)	1723 (90.9)	199 (87.3)	
*Race/ethnicity, n (%)*						< 0.001
Non-Hispanic White	12,601 (77.0)	8881 (81.1)	1936 (59.0)	1602 (84.5)	182 (79.8)	
Non-Hispanic Black	1636 (10.0)	915 (8.4)	558 (17.0)	137 (7.2)	26 (11.4)	
Other	2124 (13.0)	1161 (10.6)	787 (24.0)	156 (8.2)	20 (8.8)	
*Education, n (%)*						< 0.001
Less than college	1955 (11.9)	1100 (10.0)	630 (19.2)	194 (10.2)	31 (13.6)	
Some college	5451 (33.3)	3513 (32.1)	1304 (39.7)	550 (29.0)	84 (36.8)	
College graduate or higher	8955 (54.7)	6344 (57.9)	1347 (41.1)	1151 (60.7)	113 (49.6)	
*Employment*						< 0.001
Not employed	10,502 (64.2)	6662 (60.8)	2134 (65.0)	1522 (80.3)	184 (80.7)	
Employed	5859 (35.8)	4295 (39.2)	1147 (35.0)	373 (19.7)	44 (19.3)	
*Health insurance, n (%)*						< 0.001
Uninsured	644 (3.9)	321 (2.9)	243 (7.4)	59 (3.1)	21 (9.2)	
Insured	15,717 (96.1)	10,636 (97.1)	3038 (92.6)	1836 (96.9)	207 (90.8)	
*Household income, n (%)*						< 0.001
< $50,000	5250 (32.1)	3121 (28.5)	1558 (47.5)	507 (26.8)	64 (28.1)	
$50,000–$99,999	5665 (34.6)	3796 (34.6)	1042 (31.8)	722 (38.1)	105 (46.1)	
≥ $100,000	5446 (33.3)	4040 (36.9)	681 (20.8)	666 (35.1)	59 (25.9)	
*Marital status, n (%)*						< 0.001
Married/living with partner	10,741 (65.7)	7598 (69.3)	1616 (49.3)	1374 (72.5)	153 (67.1)	
Never married/widowed	2495 (15.2)	1567 (14.3)	650 (19.8)	246 (13.0)	32 (14.0)	
Divorced/separated	3125 (19.1)	1792 (16.4)	1015 (30.9)	275 (14.5)	43 (18.9)	
*SDOH burden (%)*						< 0.001
Low burden	8599 (52.6)	6397 (58.4)	935 (28.5)	1180 (62.3)	87 (38.2)	
High burden	7762 (47.4)	4560 (41.6)	2346 (71.5)	715 (37.7)	141 (61.8)	

Participants with cancer and PTSD (*n* = 228, 1.4%) were older than the non-cancer/non-PTSD and PTSD-only individuals (mean age: 69.5 vs. 62.9 vs. 54.4 years, respectively) and more predominantly male (*n* = 199, 87.3%) than PTSD-only (*n* = 2461, 75.0%) and non-cancer/non-PTSD (*n* = 8684, 79.3%) individuals (Table 1). On unadjusted analysis (Supplementary Table 4), compared with the control population, PTSD was associated with higher odds of healthcare utilization including inpatient visits (OR 2.48, 95% CI 2.30–2.68), emergency visits (OR 4.76, 95% CI 4.38–5.18), and other visit types (OR 4.28, 95% CI 3.87–4.72) (all *p* < 0.001). PTSD was also associated with worse overall well-being with the strongest effects related to mental (OR 6.13, 95% CI 5.62–6.69) and emotional (OR 6.13, 95% CI 5.61–6.71) health (both *p* < 0.001). A high SDoH burden was similarly associated with higher odds of emergency visits (OR 2.13, 95% CI 2.01–2.27), and increased odds of poor self-reported wellness outcomes including quality of life (OR 6.70, 95% CI 5.93–7.57) and emotional health (OR 4.54, 95% CI 4.11–5.00) (all *p* < 0.001). A cancer diagnosis itself was associated with higher odds of inpatient (OR 2.90, 95% CI 2.65–3.18) and other visit types (OR 2.32, 95% CI 2.08–2.58) (both *p* < 0.001).

### Independent impact of PTSD, cancer, and high burden SDoH on outcomes

**Fig. 2:**
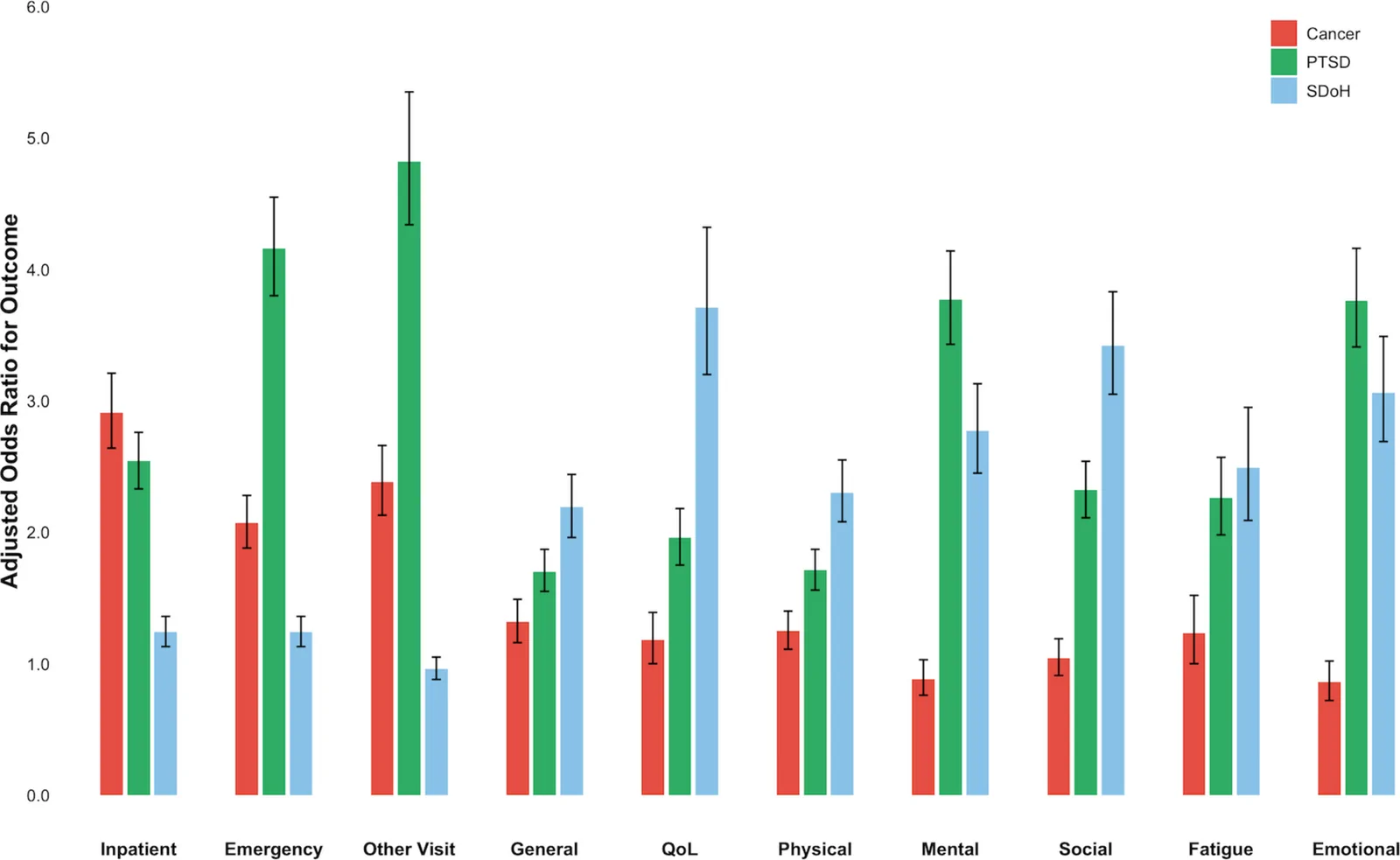
Adjusted odds ratio (with 95% CI) for inpatient, emergency, and other visit types (Home, Telehealth, Laboratory, Case management, Pharmacy), and poor health outcomes independently associated with PTSD, cancer, and high burden social determinants of health (SDoH). Inpatient, inpatient visit; emergency, emergency visit; other visit, other visit types; general, general health rating; QoL, quality of life rating; Physical, physical health rating; Mental, mental health rating; Social, social satisfaction rating; Fatigue, fatigue rating; Emotional, emotional health rating

On multivariable analyses after adjusting for sociodemographic covariates (Table 2), PTSD and SDoH remained independently associated with increased healthcare utilization and worse self-reported healthcare outcomes. Specifically, PTSD was associated with more than fourfold higher odds of emergency room visits (aOR 4.16, 95% CI 3.80–4.55) and other visit types (aOR 4.82, 95% CI 4.34–5.35). PTSD had a strong independent effect on patient self-reported poor mental (aOR 3.77, 95% CI 3.43–4.14) and emotional (aOR 3.76, 95% CI 3.41–4.16) health (both *p* < 0.001). SDoH burden was also independently associated with increased risk of inpatient (aOR 1.24, 95% CI 1.13–1.36) and emergency room (aOR 1.24, 95% CI 1.13–1.36) visits (both *p* < 0.001). Across patient-reported outcomes, high SDoH burden demonstrated consistently strong associations with poor well-being (Fig. 2). In several domains—including quality of life, social satisfaction, and emotional health—the magnitude of association for SDoH exceeded that observed for cancer diagnosis alone and approached or rivaled that of PTSD. High SDoH burden was associated with higher odds of poor quality of life (aOR 3.71, 95% CI 3.20–4.32), physical health (aOR 2.30, 95% CI 2.08–2.55), social satisfaction (aOR 3.42, 95% CI 3.05–3.83), fatigue (aOR 2.49, 95% CI 2.09–2.95), and emotional health (aOR 3.06, 95% CI 2.69–3.49) (all *p* < 0.001). Figure 2 further highlights these contrasts by visually demonstrating that SDoH burden exerted a broad, cross-domain impact on patient-reported outcomes, whereas PTSD demonstrated particularly pronounced effects on mental and emotional health, and cancer diagnosis alone was associated with comparatively smaller effect sizes. Among the five SDoH domains, economic instability and social and community context were the strongest factors driving adverse outcomes (Supplementary Table 5). Economic instability was associated with higher odds of inpatient (aOR 1.47, 95% CI 1.31–1.64) and emergency room (aOR 1.39, 95% CI 1.25–1.54) visits, as well as worse general health (aOR 2.00, 95% CI 1.76–2.28), quality of life (aOR 2.36, 95% CI 1.98–2.82), fatigue (aOR 2.05, 95% CI 1.67–2.51), and emotional health (aOR 2.04, 95% CI 1.75–2.38) (all *p* < 0.001). Neighborhood disadvantage was associated with increased emergency visits (aOR 1.15, 95% CI 1.07–1.23) and poor social satisfaction (aOR 1.36, 95% CI 1.24–1.50), while educational barriers were associated with poor physical health (aOR 1.72, 95% CI 1.43–2.06), fatigue (aOR 1.57, 95% CI 1.16–2.14), and emotional health (aOR 1.36, 95% CI 1.07–1.71) (all *p* < 0.001).

**Table 2:** Multivariable logistic regression models showing independent impacts of PTSD, cancer, and SDoH on outcomes

Outcome	PTSD		Cancer		SDoH	
	aOR (95%CI)	*p*-value	aOR (95%CI)	*p*-value	aOR (95%CI)	*p*-value
Inpatient visits*	2.54 (2.33–2.76)	< 0.001	2.91 (2.64–3.21)	< 0.001	1.24 (1.13–1.36)	< 0.001
Emergency visits*	4.16 (3.80–4.55)	< 0.001	2.07 (1.88–2.28)	< 0.001	1.24 (1.13–1.36)	< 0.001
Other visit types*	4.82 (4.34–5.35)	< 0.001	2.38 (2.13–2.66)	< 0.001	0.96 (0.88–1.05)	0.399
General health rating#	1.70 (1.55–1.87)	< 0.001	1.32 (1.16–1.49)	< 0.001	2.19 (1.96–2.44)	< 0.001
Quality of life rating#	1.96 (1.75–2.18)	< 0.001	1.18 (1.00–1.39)	0.0524	3.71 (3.20–4.32)	< 0.001
Physical health rating#	1.71 (1.56–1.87)	< 0.001	1.25 (1.11–1.40)	< 0.001	2.30 (2.08–2.55)	< 0.001
Mental health rating#	3.77 (3.43–4.14)	< 0.001	0.88 (0.76–1.03)	0.116	2.77 (2.45–3.13)	< 0.001
Social satisfaction rating#	2.32 (2.11–2.54)	< 0.001	1.04 (0.91–1.19)	0.553	3.42 (3.05–3.83)	< 0.001
Average fatigue rating+	2.26 (1.98–2.57)	< 0.001	1.23 (1.00–1.52)	0.0486	2.49 (2.09–2.95)	< 0.001
Emotional health rating+	3.76 (3.41–4.16)	< 0.001	0.86 (0.72–1.02)	0.0794	3.06 (2.69–3.49)	< 0.001

All the models included PTSD, Cancer, SDoH and were adjusted for Age, Sex, Marital status, Insurance, Race/Ethnicity, Education, Employment, and Income. The reference group for the PTSD and cancer was PTSD (no) and Cancer (no), and the reference group for the SDoH was low burden SDoH

*Variables were coded as follows: 1 (Present); Outpatient Visits, Inpatient Visits, Emergency Room visits, Ambulatory Visits, Other Visits (Home, Telehealth, Laboratory, Case management, Pharmacy)

#Self-reported ratings were recoded as binary variables: good (excellent/very good/good) vs. poor (fair/poor)

+Self-reported ratings were recoded as binary variables: good (none/never/mild/rarely/sometimes) vs. poor (very severe/always/severe/often/moderate)

Interestingly, a cancer diagnosis in the absence of PTSD or high SDoH had a more modest effect on health care utilization (emergency aOR 2.07, 95% CI 1.88–2.28; other visits aOR 2.38, 95% CI 2.13–2.66), as well as general (aOR 1.32, 95% CI 1.16–1.49) and physical (aOR 1.25, 95% CI 1.11–1.40) health (*p* < 0.001). Notably, cancer alone was not independently associated with worse mental or emotional health outcomes, underscoring the comparatively larger contribution of PTSD and SDoH to psychosocial well-being.

### Interaction and joint effects of PTSD, SDoH, and a cancer diagnosis

Joint and interaction models (Table 3) demonstrated that cancer, PTSD, and high SDoH burden were each independently associated with greater healthcare utilization and worse self-reported outcomes. Of note, exposure to a combination of these factors further amplified the risks of adverse outcomes. Patients with both cancer and PTSD exhibited the highest acute healthcare utilization, with markedly elevated odds of emergency department visits (aOR 6.45, 95% CI 4.68–8.88) and inpatient admissions (aOR 4.42, 95% CI 3.36–5.80) with strong evidence of interaction (*p* < 0.001 and *p* = 0.006, respectively) compared with controls. For other visit types and most self-reported outcomes, joint effects were elevated but without multiplicative interaction, consistent with additive risk patterns.

**Table 3:** Multivariable logistic regression models showing interaction and joint effects of PTSD, cancer, and SDoH on the outcomes

Panel A. PTSD × Cancer				
Outcome	PTSD only aOR (95% CI)	Cancer only aOR (95% CI)	Joint effect aOR (95% CI)	Interaction *p*-value
Inpatient visits	2.87 (2.62–3.14)	3.38 (3.05–3.75)	4.42 (3.36–5.80)	< 0.001
Emergency visits	4.45 (4.06–4.89)	2.34 (2.11–2.59)	6.45 (4.68–8.88)	0.0059
Other visit types	4.94 (4.44–5.50)	2.55 (2.27–2.85)	20.01 (10.25–39.03)	0.183
General health rating	1.72 (1.56–1.90)	1.37 (1.20–1.57)	2.35 (1.75–3.14)	0.977
Quality of life rating	1.93 (1.72–2.16)	1.19 (0.98–1.43)	3.00 (2.15–4.19)	0.168
Physical health rating	1.72 (1.56–1.88)	1.29 (1.13–1.46)	2.32 (1.75–3.07)	0.765
Mental health rating	3.79 (3.43–4.18)	1.01 (0.84–1.21)	3.61 (2.67–4.90)	0.760
Social satisfaction rating	2.33 (2.12–2.57)	1.12 (0.97–1.30)	2.50 (1.86–3.38)	0.799
Fatigue rating	2.28 (1.98–2.61)	1.33 (1.05–1.69)	3.12 (2.08–4.68)	0.898
Emotional health rating	3.76 (3.39–4.17)	0.97 (0.79–1.19)	3.68 (2.67–5.07)	0.971
Panel B. Cancer × SDoH				
Outcome	Cancer only aOR (95% CI)	SDoH only aOR (95% CI)	Joint effect aOR (95% CI)	Interaction *p*-value
Inpatient visits	3.14 (2.77–3.56)	1.24 (1.13–1.38)	3.85 (3.28–4.52)	0.885
Emergency visits	2.15 (1.90–2.44)	1.22 (1.11–1.34)	2.97 (2.52–3.50)	0.220
Other visit types	2.14 (1.87–2.46)	0.91 (0.83–1.00)	3.32 (2.72–4.06)	< 0.001
General health rating	1.51 (1.26–1.81)	2.25 (2.00–2.53)	2.84 (2.37–3.41)	0.139
Quality of life rating	1.23 (0.90–1.68)	3.70 (3.15–4.33)	4.70 (3.74–5.90)	0.853
Physical health rating	1.42 (1.20–1.69)	2.36 (2.12–2.64)	2.83 (2.38–3.36)	0.132
Mental health rating	0.98 (0.76–1.28)	2.77 (2.43–3.14)	2.77 (2.23–3.43)	0.925
Social satisfaction rating	1.06 (0.85–1.32)	3.39 (3.01–3.81)	3.87 (3.21–4.67)	0.597
Fatigue rating	1.33 (0.95–1.87)	2.48 (2.07–2.97)	3.33 (2.50–4.44)	0.975
Emotional health rating	0.73 (0.53–1.01)	2.95 (2.57–3.37)	3.27 (2.61–4.11)	0.028
Panel C. PTSD × SDoH				
Outcome	PTSD only aOR (95% CI)	SDoH only aOR (95% CI)	Joint effect aOR (95% CI)	Interaction *p*-value
Inpatient visits	2.30 (2.00–2.66)	1.18 (1.07–1.31)	3.46 (3.05–3.92)	0.006
Emergency visits	4.66 (4.04–5.38)	1.26 (1.15–1.39)	5.21 (4.58–5.92)	0.178
Other visit types	8.72 (7.04–10.79)	1.03 (0.94–1.14)	4.14 (3.60–4.75)	< 0.001
General health rating	2.50 (2.11–2.97)	2.46 (2.18–2.77)	3.69 (3.20–4.25)	< 0.001
Quality of life rating	3.34 (2.62–4.25)	4.45 (3.74–5.28)	7.87 (6.52–9.50)	< 0.001
Physical health rating	2.57 (2.19–3.02)	2.60 (2.33–2.91)	3.85 (3.36–4.40)	< 0.001
Mental health rating	6.53 (5.51–7.75)	3.61 (3.12–4.16)	10.92 (9.33–12.77)	< 0.001
Social satisfaction rating	3.18 (2.68–3.77)	3.82 (3.37–4.33)	7.95 (6.88–9.18)	< 0.001
Fatigue rating	3.32 (2.58–4.26)	2.91 (2.39–3.54)	5.89 (4.76–7.29)	< 0.001
Emotional health rating	6.18 (5.15–7.42)	3.91 (3.35–4.56)	12.20 (10.33–14.41)	< 0.001

Panel A: Joint aOR compares PTSD and Cancer vs Control; interaction *p* tests departure from multiplicativity. Joint effects (Cancer + PTSD vs control) were obtained by exponentiating the sum of the PTSD, cancer, and interaction coefficients: aORPTSD + Cancer = exp(βPTSD + βCancer + βPTSD × Cancer) [30]. Models adjusted for age, sex, race/ethnicity, education, employment, insurance, income, marital status, and SDoH burden. Panel B: Joint aOR compares Cancer and high burden SDoH vs Control; interaction *p* tests departure from multiplicativity. Joint effects (Cancer + high burden SDoH vs control) were obtained by exponentiating the sum of the cancer, high burden SDoH, and interaction coefficients: aORCancer + SDoH = exp(βCancer + βSDoH + βCancer × SDoH) [30]. Models adjusted for age, sex, race/ethnicity, education, employment, insurance, income, marital status, and PTSD. Panel C: Joint aOR compares PTSD and high burden SDoH vs Control; interaction p tests departure from multiplicativity. Joint effects (PTSD + high burden SDoH vs control) were obtained by exponentiating the sum of the PTSD, SDoH, and interaction coefficients: aORPTSD + SDoH = exp(βPTSD + βSDoH + βPTSD × SDoH) [30]. Models adjusted for age, sex, race/ethnicity, education, employment, insurance, income, marital status, and cancer

**Fig. 3:**
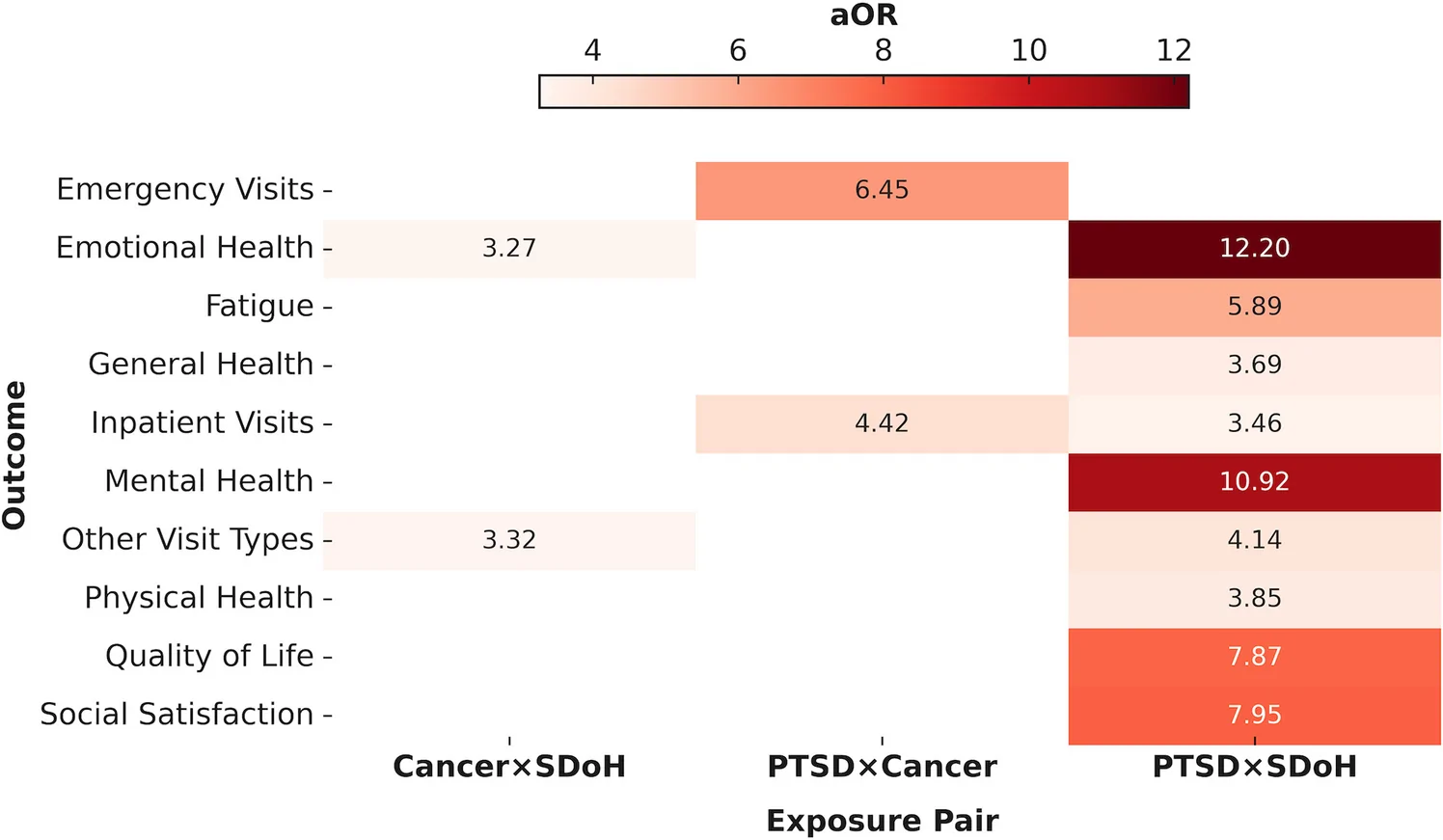
Heatmap of adjusted odds ratios (aOR) for significant interaction effects related to combined exposures and outcomes

The combination of cancer and high SDoH burden also affected patient outcomes. Joint effects were observed for other visit types (aOR 3.32, 95% CI 2.72–4.06) and poor emotional health (aOR 3.27, 95% CI 2.61–4.11), with multiplicative interaction for both outcomes (*p* < 0.001 and *p* = 0.028, respectively). The joint impact of PTSD and high SDoH among cancer patients was most prominent and impacted both healthcare utilization and psychosocial outcomes (Table 3). Cancer patients with both PTSD and high SDoH burden had substantially greater odds of poor mental health (aOR 10.92, 95% CI 9.33–12.77), poor emotional health (aOR 12.20, 95% CI 10.33–14.41), diminished quality of life (aOR 7.87, 95% CI 6.52–9.50), and low social satisfaction (aOR 7.95, 95% CI 6.88–9.18) (all *p* < 0.001). Interactions were highly significant across these domains (all *p* < 0.001), underscoring that PTSD and SDoH exerted synergistic effects on cancer patient outcomes (Fig. 3).

## Discussion

Within the Healthy People 2030 framework, social determinants of health (SDoH) are recognized as modifiable drivers of cancer outcomes alongside biology and care delivery [14, 15]. Cancer itself can act as a traumatic stressor, with cancer-related PTSD emerging as a survivorship challenge that worsens distress and outcomes [3, 4, 9]. The current study was important as we examined a large national cohort using the All of Us Research Program, which linked electronic health record diagnoses with self-reported patient survey data. Interestingly, cancer, PTSD, and SDoH each were independently associated with healthcare utilization and self-reported health outcomes among cancer survivors. PTSD was associated with markedly higher acute healthcare utilization including emergency and inpatient visits, as well as substantially worse mental and emotional health. Furthermore, a high SDoH burden predicted poor quality of life, social satisfaction, fatigue, and emotional well-being among cancer survivors, highlighting the dominant role that social factors play in shaping patient-reported outcomes. While associated with higher odds of certain health-care visits, a cancer diagnosis alone had less of an independent impact on the general, as well as physical and mental health of cancer survivors. Rather, joint exposures amplified the likelihood of adverse outcomes with concurrent PTSD and high SDoH conferring the greatest risk of inpatient and emergency visits and worse self-reported health outcomes among cancer survivors compared with the general population.

Cancer-related PTSD is generally characterized by hyperarousal, intrusive re-experiencing, avoidance, and dysregulated emotion [3–5, 31, 32]. These symptoms can undermine sleep quality and intensify pain perception among cancer survivors, as well as interfere with adherence to therapy [4, 33]. Such disruptions, in turn, predispose patients to crisis-oriented care, which could explain why PTSD emerged as a strong independent predictor of emergency and inpatient acute care utilization in the current study. Other reports have similarly noted a substantially higher hospitalization and emergency room use among non-cancer adults with PTSD, even after adjustment for comorbidity [34]; one study based on civilian US population reported increased emergency and inpatient visits among individuals with versus without PTSD [22]. In addition to PTSD, we also noted that a high burden of adverse SDoH was strongly associated with worse well-being among cancer survivors. Although the strength of the association varied across individual SDoH domains, a key observation was that social determinants exerted a relatively stronger influence than PTSD alone on patient-reported outcomes (Table 2 and Fig. 2). Population-based evidence has indicated that cumulative social disadvantage—including economic instability, underinsurance, limited education, neighborhood deprivation, and social isolation—strongly shapes both mortality and survivorship among US cancer survivors [15, 17, 18, 35–37]. Taken together, these findings highlight the interactive impact of cancer, PTSD, and social adversity as critical drivers of survivorship outcomes. In turn, the data underscore the importance of integrating social and environmental context into the diagnosis, treatment, and long-term support of cancer survivors living with PTSD.

In examining the various SDoH domains, economic instability demonstrated the strongest and most pervasive association with worse outcomes across the cohort (Supplementary Table 5). Other recent data have similarly reported that the strong connection of financial toxicity with inferior health-related quality of life, care disruptions, and—even in resource-rich settings—worse survival [37, 38]. Social and community context—which captured features of isolation, low support, and discrimination—was likewise consequential for mental and social health; contemporary studies have implicated social isolation as a potent determinant of anxiety, depression, and lower quality of life in cancer survivorship [39–41]. In the current study, neighborhood disadvantage related to greater emergency use and worse social satisfaction among cancer survivors, which has been noted to impact cancer-specific survival [15, 36]. In addition, healthcare access/quality and educational barriers were associated with physical and fatigue outcomes, consistent with work demonstrating that transportation constraints and fragmented access drive missed care, emergency reliance, and symptom burden among cancer survivors [36, 42–45]. These findings identify social risk as a modifiable driver of disparities—underscoring that equity-focused interventions targeting financial hardship, social support, and access barriers could yield meaningful improvements in well-being and outcomes among individuals living with cancer.

One particular strength of the current study was the use of joint and interaction models to demonstrate the combinatorial effect of cancer, PTSD, and SDoH on outcomes. In fact, a cancer diagnosis combined with PTSD dramatically increased emergency and inpatient utilization (Table 3); in contrast, the interaction of PTSD with SDoH had a strong impact on patient-reported health outcomes. Cancer-related PTSD symptoms can correlate with substantially greater symptom burden often requiring greater use of healthcare resources [46]; furthermore, adverse SDoH often shape patient-reported outcomes such as overall well-being and perception of wellness among cancer patients [47]. The interaction of PTSD and SDoH with a cancer diagnosis to increase the risk of adverse outcomes underlines the urgency for an integrated oncology care plan that addresses both PTSD and the social factors in the survivorship setting. Routine and repeat PTSD screening embedded in oncology care (e.g., at diagnosis, treatment transitions, and survivorship) with rapid access to evidence-based trauma therapies and collaborative mental-health care have demonstrated benefit in trauma-related care [48–50]. In parallel, health systems should normalize SDoH screening and improve responses to identified needs (e.g., financial strain, food insecurity, transportation, insurance navigation, social connection) with on-site navigation and community partnerships—an approach explicitly endorsed in national policy statements from ASCO [51]. Financial toxicity, underinsurance, and travel burdens restrict access to preventive and supportive services and erode social buffering, amplifying fatigue, emotional distress, and diminished quality of life [51, 52]. Moreover, the finding that a cancer diagnosis itself had less of an independent association with mental and emotional outcomes after accounting for PTSD and SDoH suggested that, for some survivors, their lived experience might be shaped less by cancer itself and more by psychological trauma and the social context related to surviving with a cancer diagnosis. This observation is consistent with contemporary survivorship science and with emerging psychiatric frameworks that extend DSM-5 trauma criteria to medical illness, underscoring that these psychosocial and structural determinants—rather than the malignancy itself—frequently drive mental and emotional well-being [5, 19, 36, 37, 47, 53]. In turn, care providers need to prioritize proactive outreach, same-day supportive visits, and after-hours access for individuals with concurrent PTSD and high SDoH burden to improve well-being [48, 50, 54].

The current study had several limitations. Although providing scale and diversity, the All of Us cohort was not a probability sample and thus generalizability to the broader US population may be limited. Ascertainment of PTSD and cancer diagnoses through EHR codes may have introduced potential misclassification and imprecision relative to survey responses, while the cross-sectional nature of the psychosocial measures constrained causal inference. Residual confounding from factors such as pain, treatment intensity, and multimorbidity also could not be excluded. In addition, the necessary dichotomization of SDoH domains and patient-reported outcomes may have attenuated gradients and masked dose–response relationships. Future studies should employ population-representative, longitudinal designs with validated measures of PTSD, cancer, and psychosocial constructs to strengthen causal inference and reduce misclassification.

In conclusion, among individuals with cancer, PTSD and adverse SDoH each imposed a substantial and combinatorial burden on cancer survivors. PTSD was a key driver of increased acute care utilization, while social adversity had a larger impact on psychosocial well-being. Patients who suffered from both PTSD and a high SDoH had the greatest risk of adverse outcomes including higher healthcare utilization and worse self-reported health outcomes. Survivorship care should incorporate early detection and treatment of PTSD, as well as accessible social support services and proactive measures to address SDoH. Embedding these strategies into routine cancer care practice has the potential to transform survivorship and improve patient experience while advancing equity and reducing healthcare utilization.

## Supplementary Information

Below is the link to the electronic supplementary material.

ESM 1 (DOCX 971 KB)

## Data Availability

Data availability is based on the All of Us Research Program Data policy.
