# Supplementary material for: Post-traumatic stress disorder and social determinants of health among individuals with cancer: association with healthcare utilization and outcomes in the All of Us Research Program
Source: J Cancer Surviv. 2026 Mar 3;20(Suppl 2):1636–48. doi: 10.1007/s11764-026-01993-8 (PMC13744465; doi:10.1007/s11764-026-01993-8)
Supplement: Supplementary file 1 [file 11764_2026_1993_MOESM1_ESM.docx]

**Supplementary Material**

**Supplementary Figure 1.** Conceptual Framework Linking Cancer, PTSD, Social Determinants of Health, and healthcare utilization and patient-reported outcomes

Note: Arrows indicate hypothesized relationships between cancer, PTSD, SDoH, and healthcare utilization and patient-reported outcomes

**Supplementary Table 1.** Social Determinant of Health Survey Questions and Domains

| Neighborhood and Built Environment | | | | |  |
| --- | --- | --- | --- | --- | --- |
| N1 | People around here are willing to help their neighbors. | | | |  |
| N2 | People in my neighborhood generally get along with each other. | | | |  |
| N3 | People in my neighborhood can be trusted. | | | |  |
| N4 | People in my neighborhood share the same values. | | | |  |
| N5 | There is a lot of graffiti in my neighborhood. | | | |  |
| N6 | My neighborhood is noisy. | | | |  |
| N7 | Vandalism is common in my neighborhood. | | | |  |
| N8 | There are a lot of abandoned buildings in my neighborhood. | | | |  |
| N9 | My neighborhood is clean. | | | |  |
| N10 | People in my neighborhood take good care of their houses and apartments. | | | |  |
| N11 | There are too many people hanging around on the streets near my home. | | | |  |
| N12 | There is a lot of crime in my neighborhood. | | | |  |
| N13 | There is too much drug use in my neighborhood. | | | |  |
| N14 | There is too much alcohol use in my neighborhood. | | | |  |
| N15 | I’m always having trouble with my neighbors. | | | |  |
| N16 | In my neighborhood, people watch out for each other. | | | |  |
| N17 | My neighborhood is safe. | | | |  |
| N18 | Many shops, stores, markets or other places to buy things I need are within easy walking distance of my home. Would you say that you… | | | |  |
| N19 | It is within a 10-15 minute walk to a transit stop (such as bus, train, trolley, or tram) from my home. Would you say that you… | | | |  |
| N20 | There are sidewalks on most of the streets in my neighborhood. Would you say that you… | | | |  |
| N21 | There are facilities to bicycle in or near my neighborhood, such as special lanes, separate paths or trails, shared use paths for cycles and pedestrians. Would you say that you… | | | |  |
| N22 | The crime rate in my neighborhood makes it unsafe to go on walks at night. Would you say that you… | | | |  |
| N23 | The crime rate in my neighborhood makes it unsafe to go on walks during the day. Would you say that you… | | | |  |
|  |  | | | |  |
| Social and Community Context | | | | |  |
| S1 | Choose the answer that best describes how often you can find support if you need it. Someone to help you if you were confined to bed | | | |  |
| S2 | Choose the answer that best describes how often you can find support if you need it. Someone to take you to the doctor if you need it | | | |  |
| S3 | Choose the answer that best describes how often you can find support if you need it. Someone to help with daily chores if you were sick | | | |  |
| S4 | Choose the answer that best describes how often you can find support if you need it. Someone to have a good time with | | | |  |
| S5 | Choose the answer that best describes how often you can find support if you need it. Someone to turn to for suggestions about how to deal with a personal problem | | | |  |
| S6 | Choose the answer that best describes how often you can find support if you need it. Someone to love and make you feel wanted | | | |  |
| S7 | Choose the answer that is true for you. I lack companionship | | | |  |
| S8 | Choose the answer that is true for you. There is no one I can turn to | | | |  |
| S9 | Choose the answer that is true for you. I am an outgoing person | | | |  |
| S10 | Choose the answer that is true for you. I feel left out | | | |  |
| S11 | Choose the answer that is true for you. I feel isolated from others | | | |  |
| S12 | Choose the answer that is true for you. I can find companionship when I want it | | | |  |
| S13 | Choose the answer that is true for you. I am unhappy being so withdrawn | | | |  |
| S14 | Choose the answer that is true for you. People are around me but not with me | | | |  |
| S15 | In your day-to-day life, how often do any of these happen to you? You are treated with less respect than other people are. | | | |  |
| S16 | In your day-to-day life, how often do any of these happen to you? You receive poorer service than other people at restaurants or stores. | | | |  |
| S17 “What is your current marital status?” | | | |  |  |
|  | | | |  |  |
| Economic Stability | | | | |  |
| ES1 “What is your annual household income from all sources?” | | | | |  |
| ES2 Area Deprivation index: A deprivation index for each census tract in the US based on six different  ACS measures. The index ranges from 0 to 1, with a higher index being more deprived. | | | | |  |
| ES3 “What is your current employment status?” | |  | | |  |
| ES4 | Within the past 12 months, the food we bought just didn’t last and we didn’t have money to get more. | | | |  |
| ES5 | In the last 12 months, how many times have you or your family moved from one home to another? Number of moves in the past 12 months: | | | |  |
|  |  | | | |  |
|  | Education Access and Quality | | | |  |
| “What is the highest grade or year of school you completed?” | | |  | |  |
|  |  | | | |  |
| Healthcare Access and Quality | | | | |  |
| HAQ 1 “Do you have health insurance coverage?” | | | | |  |
| HAQ2 “What kind of health insurance or health care coverage do you have?” | | | | |  |
| HAQ3 | “DURING THE PAST 12 MONTHS, were you told by a health care provider or doctor’s office that they did not accept your health care coverage?” | | | |  |
| HAQ4 | About how long has it been since you last saw or talked to a doctor or other health care professional about your own health? | | | |  |
| HAQ5 | There are many reasons people delay getting medical care. Have you delayed getting care for any of the following reasons in the PAST 12 MONTHS? Didn’t have transportation. | | | |  |
| HAQ6 | There are many reasons people delay getting medical care. Have you delayed getting care for any of the following reasons in the PAST 12 MONTHS? You live in a rural area where distance to the health care provider is too far. | | | |  |
| HAQ7 | There are many reasons people delay getting medical care. Have you delayed getting care for any of the following reasons in the PAST 12 MONTHS? Couldn’t get time off work. | | | |  |
| HAQ8 | There are many reasons people delay getting medical care. Have you delayed getting care for any of the following reasons in the PAST 12 MONTHS? Couldn’t get child care. | | | |  |
| HAQ9 | There are many reasons people delay getting medical care. Have you delayed getting care for any of the following reasons in the PAST 12 MONTHS? Couldn’t afford the copay. | | | |  |
| HAQ10 | There are many reasons people delay getting medical care. Have you delayed getting care for any of the following reasons in the PAST 12 MONTHS? Your deductible was too high/or could not afford the deductible. | | | |  |
| HAQ11 | There are many reasons people delay getting medical care. Have you delayed getting care for any of the following reasons in the PAST 12 MONTHS? You had to pay out of pocket for some or all of the procedure. | | | |  |
| HAQ12 | DURING THE PAST 12 MONTHS, was there any time when you needed any of the following, but didn’t get it because you couldn’t afford it? Prescription medicines | | | |  |
| HAQ13 | DURING THE PAST 12 MONTHS, was there any time when you needed any of the following, but didn’t get it because you couldn’t afford it? Mental health care or counseling | | | |  |
| HAQ14 | DURING THE PAST 12 MONTHS, was there any time when you needed any of the following, but didn’t get it because you couldn’t afford it? Emergency care | | | |  |
| HAQ15 | DURING THE PAST 12 MONTHS, was there any time when you needed any of the following, but didn’t get it because you couldn’t afford it? To see a regular doctor or general health provider (in primary care, general practice, internal medicine, family medicine) | | | |  |
| HAQ16 | DURING THE PAST 12 MONTHS, was there any time when you needed any of the following, but didn’t get it because you couldn’t afford it? To see a specialist | | | |  |
| HAQ17 | DURING THE PAST 12 MONTHS, were any of the following true for you: You skipped medication doses to save money | | | |  |
| HAQ18 | DURING THE PAST 12 MONTHS, were any of the following true for you: You took less medicine to save money | | | |  |
| HAQ19 | DURING THE PAST 12 MONTHS, were any of the following true for you: You delayed filling a prescription to save money | | | |  |
| HAQ20 | DURING THE PAST 12 MONTHS, were any of the following true for you: You asked your doctor for a lower cost medication to save money | | | |  |
| HAQ21 | DURING THE PAST 12 MONTHS, were any of the following true for you: You used alternative therapies to save money | | | |  |

**Supplementary Table 2.** Survey Questions and Response Types on Self-reported well-being Outcomes

| **Category** | Questions | Response |
| --- | --- | --- |
|  |  |  |
| **General Health** | In general, would you say your health is: | Categorical (Ordinal)  *(1-Poor, 2-Fair, 3-Good, 4-Very Good,*  *5-Excellent)* |
| **Quality of Life** | In general, would you say your quality of life is: | Categorical (Ordinal)  *(1-Poor, 2-Fair, 3-Good, 4-Very Good,*  *5-Excellent)* |
| **Physical Health** | In general, how would you rate your physical health? | Categorical (Ordinal)  *(1-Poor, 2-Fair, 3-Good, 4-Very Good,*  *5-Excellent)* |
| **Mental Health** | In general, how would you rate your mental health, including your mood and your ability to think? | Categorical (Ordinal)  *(1-Poor, 2-Fair, 3-Good, 4-Very Good,*  *5-Excellent)* |
| **Social Satisfaction** | In general, how would you rate your satisfaction with your social activities and relationships? | Categorical (Ordinal)  *(1-Poor, 2-Fair, 3-Good, 4-Very Good,*  *5-Excellent)* |
| **Fatigue Rating** | In the past 7 days, how would you rate your fatigue on average? | Categorical (Ordinal)  *(1-Very Severe, 2-Severe, 3-Moderate,*  *4-Mild, 5-None)* |
| **Emotional Health** | In the past 7 days, how often have you been bothered by emotional problems such as feeling anxious, depressed or irritable? | Categorical (Ordinal)  *(1-Always, 2-Often, 3-Sometimes,*  *4-Rarely, 5-Never)* |

**Supplementary Table 3**. Frequency Distribution of Outcomes (Outcome = 1) across the Exposure Groups

| Outcome | No Cancer No PTSD Cohort (Control) (n=10,957) | PTSD Only Cohort  (n=3,281) | Cancer Only Cohort  (n=1,895) | Cancer and PTSD Cohort (n=228) |
| --- | --- | --- | --- | --- |
| Outpatient Visits | 10,935 (99.8%) | 3,281 (100.0%) | 1,893 (99.9%) | 227 (99.6%) |
| Inpatient Visits | 2,597 (23.7%) | 1,587 (48.4%) | 1,021 (53.9%) | 141 (61.9%) |
| Emergency Visits | 3,812 (34.8%) | 2,428 (74.0%) | 1,035 (54.6%) | 179 (78.4%) |
| Ambulatory Visits | 581 (5.3%) | 131 (4.0%) | 176 (9.3%) | 4 (1.7%) |
| Other Visit Types | 5,896 (53.8%) | 2,766 (84.3%) | 1,442 (76.1%) | 219 (96.1%) |
| General Health Rating | 1,687 (15.4%) | 1,064 (32.4%) | 341 (18.0%) | 73 (32.0%) |
| Quality of Life Rating | 932 (8.5%) | 830 (25.3%) | 152 (8.0%) | 52 (22.9%) |
| Physical Health Rating | 2,037 (18.6%) | 1,237 (37.7%) | 388 (20.5%) | 84 (36.8%) |
| Mental Health Rating | 1,227 (11.2%) | 1,425 (43.4%) | 152 (8.0%) | 66 (29.0%) |
| Social Satisfaction Rating | 1,655 (15.1%) | 1,322 (40.3%) | 256 (13.5%) | 72 (31.6%) |
| Average Fatigue Rating | 548 (5.0%) | 532 (16.2%) | 87 (4.6%) | 30 (13.0%) |
| Emotional Health Rating | 1,064 (9.7%) | 1,289 (39.3%) | 118 (6.2%) | 57 (25.1%) |

Values are presented as number (percentage) of participants with outcome = 1 within each group. Outpatient visits (99.9% with ≥1 visit; n=21 with none) and ambulatory visits (very low counts in some groups) showed insufficient variation for regression analysis.

**Supplementary Table 4**. Unadjusted Logistic regression Models Showing the Odds Ratio (OR) of the Association of PTSD, Cancer, and SDoH burden with Healthcare Utilization and Poor Self-reported Health Outcomes

| Outcome | PTSD | | Cancer | | SDoH | |
| --- | --- | --- | --- | --- | --- | --- |
|  | OR (95% CI) | p-value | OR (95% CI) | p-value | OR (95% CI) | p-value |
|  |  |  |  |  |  |  |
| Inpatient Visits* | 2.48 (2.30–2.68) | <0.001 | 2.90 (2.65–3.18) | <0.001 | 1.72 (1.61–1.83) | <0.001 |
| Emergency Visits* | 4.76 (4.38–5.18) | <0.001 | 1.71 (1.56–1.88) | <0.001 | 2.13 (2.01–2.27) | <0.001 |
| Other Visit Types* | 4.28 (3.87–4.72) | <0.001 | 2.32 (2.08–2.58) | <0.001 | 1.32 (1.24–1.41) | <0.001 |
| General Health Rating# | 2.56 (2.35–2.78) | <0.001 | 1.02 (0.91–1.14) | 0.783 | 3.32 (3.05–3.61) | <0.001 |
| Quality of Life Rating# | 3.65 (3.31–4.02) | <0.001 | 0.75 (0.65–0.87) | 0.0002 | 6.70 (5.93–7.57) | <0.001 |
| Physical Health Rating# | 2.58 (2.38–2.80) | <0.001 | 0.96 (0.86–1.07) | 0.432 | 3.45 (3.19–3.73) | <0.001 |
| Mental Health Rating# | 6.13 (5.62–6.69) | <0.001 | 0.50 (0.43–0.58) | <0.001 | 4.38 (4.00–4.80) | <0.001 |
| Social Satisfaction Rating# | 3.78 (3.48–4.10) | <0.001 | 0.69 (0.61–0.78) | <0.001 | 4.68 (4.29–5.10) | <0.001 |
| Average Fatigue Rating+ | 3.64 (3.23–4.10) | <0.001 | 0.71 (0.59–0.87) | 0.0006 | 3.55 (3.10–4.05) | <0.001 |
| Emotional Health Rating+ | 6.13 (5.61–6.71) | <0.001 | 0.45 (0.39–0.53) | <0.001 | 4.54 (4.11–5.00) | <0.001 |

Note. *Variables were coded as follows: 1 (Present); Outpatient Visits, Inpatient Visits, Emergency Room visits, Ambulatory Visits, Other Visits (Home, Telehealth, Laboratory, Case management, Pharmacy)

\# Self-reported ratings were recoded as binary variables: good (excellent/very good/good) vs. poor (fair/poor)

+ Self-reported ratings were recoded as binary variables: good (none/never/mild/rarely/sometimes) vs. poor (very

severe/always/severe/often/moderate)

The reference group for the PTSD and cancer was PTSD (no) and Cancer (no), and the reference group for the SDoH was low burden SDoH

**Supplementary Table 5.** Multivariable Logistic Regression Models showing Adjusted Odds Ratios (aOR) with 95% Confidence Intervals for Healthcare Utilization and Poor Health Outcomes by SDoH Domains

| Outcome | Economic Stability*  #aOR (95%CI) | Education Access & Quality*  aOR (95%CI) | Healthcare Access and Quality*  aOR (95%CI) | Neighborhood and Built Environment*  aOR (95%CI) | Social and Community Context*  aOR (95%CI) |
| --- | --- | --- | --- | --- | --- |
|  |  |  |  |  |  |
| Inpatient Visits | 1.47 (1.31–1.64) | 1.03 (0.86–1.22) | 0.84 (0.78–0.91) | 1.10 (1.01–1.18) | 1.14 (1.05–1.23) |
| Emergency Visits | 1.39 (1.25–1.54) | 1.15 (0.98–1.36) | 0.75 (0.69–0.80) | 1.15 (1.07–1.23) | 1.16 (1.08–1.25) |
| Other Visit Types | 1.04 (0.93–1.15) | 1.03 (0.86–1.22) | 0.68 (0.63–0.74) | 0.89 (0.82–0.95) | 1.06 (0.99–1.15) |
| General Health Rating | 2.00 (1.76–2.28) | 1.59 (1.31–1.93) | 1.24 (1.13–1.36) | 1.24 (1.13–1.36) | 1.60 (1.45–1.76) |
| Quality of Life Rating | 2.36 (1.98–2.82) | 1.31 (1.04–1.67) | 1.34 (1.19–1.51) | 1.31 (1.16–1.48) | 2.45 (2.15–2.80) |
| Physical Health Rating | 2.00 (1.77–2.26) | 1.72 (1.43–2.06) | 1.17 (1.07–1.27) | 1.27 (1.16–1.38) | 1.66 (1.52–1.81) |
| Mental Health Rating | 2.15 (1.86–2.48) | 1.12 (0.90–1.40) | 1.21 (1.09–1.35) | 1.23 (1.11–1.37) | 2.29 (2.05–2.56) |
| Social Satisfaction Rating | 1.89 (1.66–2.17) | 1.23 (1.00–1.51) | 1.13 (1.03–1.25) | 1.36 (1.24–1.50) | 3.54 (3.19–3.94) |
| Fatigue Rating | 2.05 (1.67–2.51) | 1.57 (1.16–2.14) | 1.28 (1.11–1.48) | 1.17 (1.01–1.35) | 1.83 (1.57–2.14) |
| Emotional Health Rating | 2.04 (1.75–2.38) | 1.36 (1.07–1.71) | 1.12 (1.00–1.26) | 1.19 (1.07–1.33) | 2.19 (1.95–2.47) |

* Values represent binary domain scores, where 1 = high burden (poor conditions) and 0 = low burden.

\# Adjusted for PTSD, cancer, Age, Sex, Marital status, Insurance, Race/Ethnicity, Education, Employment and

Income
